# Mycotoxigenic Potentials of *Fusarium* Species in Various Culture Matrices Revealed by Mycotoxin Profiling

**DOI:** 10.3390/toxins9010006

**Published:** 2016-12-26

**Authors:** Wen Shi, Yanglan Tan, Shuangxia Wang, Donald M. Gardiner, Sarah De Saeger, Yucai Liao, Cheng Wang, Yingying Fan, Zhouping Wang, Aibo Wu

**Affiliations:** 1 State Key Laboratory of Food Science and Technology, School of Food Science and Technology, Jiangnan University, Wuxi 214122, China; sw1596321@sina.com; 2 SIBS-UGENT-SJTU Joint Laboratory of Mycotoxin Research, Key Laboratory of Food Safety Research, Institute for Nutritional Sciences, Shanghai Institutes for Biological Sciences, Chinese Academy of Sciences, University of Chinese Academy of Sciences, 294 Taiyuan Road, Shanghai 200031, China; yltan@sibs.ac.cn (Y.T.); shuangxiawang@163.com (S.W.); 3 Commonwealth Scientific and Industrial Research Organisation (CSIRO), 306 Carmody Road, St Lucia QLD 4067, Australia; Donald.Gardiner@csiro.au; 4 Laboratory of Food Analysis, Faculty of Pharmaceutical Sciences, Ghent University, Ottergemsesteenweg 460, Gent 9000, Belgium; Sarah.DeSaeger@UGent.be; 5 College of Plant Science and Technology, Huazhong Agricultural University, Wuhan 430000, China; yucailiao@mail.hzau.edu.cn; 6 Institute of Quality Standards & Testing Technology for Agro-Products, Laboratory of Quality and Safety Risk Assessment for Agro-Products (Urumqi), Ministry of Agriculture, Xinjiang Academy of Agricultural Sciences, 403 Nanchang Road, Urumqi 830091, China; wangcheng312@sina.com (C.W.); fyyxaas@sina.com (Y.F.)

**Keywords:** *Fusarium* fungi, mycotoxin profiles, principal component analysis, culture substrates

## Abstract

In this study, twenty of the most common *Fusarium* species were molecularly characterized and inoculated on potato dextrose agar (PDA), rice and maize medium, where thirty three targeted mycotoxins, which might be the secondary metabolites of the identified fungal species, were detected by liquid chromatography–tandem mass spectrometry (LC-MS/MS). Statistical analysis was performed with principal component analysis (PCA) to characterize the mycotoxin profiles for the twenty fungi, suggesting that these fungi species could be discriminated and divided into three groups as follows. Group I, the fusaric acid producers, were defined into two subgroups, namely subgroup I as producers of fusaric acid and fumonisins, comprising of *F. proliferatum*, *F. verticillioides*, *F. fujikuroi* and *F. solani*, and subgroup II considered to only produce fusaric acid, including *F. temperatum*, *F. subglutinans*, *F. musae*, *F. tricinctum*, *F. oxysporum*, *F. equiseti*, *F. sacchari*, *F. concentricum*, *F. andiyazi*. Group II, as type A trichothecenes producers, included *F. langsethiae*, *F. sporotrichioides*, *F. polyphialidicum*, while Group III were found to mainly produce type B trichothecenes, comprising of *F. culmorum*, *F. poae*, *F. meridionale* and *F. graminearum*. A comprehensive picture, which presents the mycotoxin-producing patterns by the selected fungal species in various matrices, is obtained for the first time, and thus from an application point of view, provides key information to explore mycotoxigenic potentials of *Fusarium* species and forecast the *Fusarium* infestation/mycotoxins contamination.

## 1. Introduction

*Fusarium* spp. are a large complex genus, known as worldwide plant pathogens which infect and colonize various cereal crops such as maize, rice, wheat and oats in temperate and semi-tropical areas, including China, North America, South Africa and all European cereal-growing areas [1,2,3,4,5]. *Fusarium* spp. have been found to cause significant reduction in quality and yield in many food and feed crops, estimated at between 10% and 30%. The worst affected crops are wheat, maize and rice, where *Fusarium* spp. are known to cause *Fusarium* head blight (FHB) of wheat, sheath rot disease of maize and bakanae disease of rice [6,7,8,9].

The widespread presence of fungi and mycotoxins in pre-harvest infected plants or in-store grains are of great concern for human and animal health. The most occurring *Fusarium* mycotoxins are deoxynivalenol (DON), 3-acetyl deoxynivalenol (3-ADON), 15-acetyl deoxynivalenol (15-ADON), nivalenol (NIV) and fusarenon X (Fus-X); T-2 toxin, HT-2 toxin, neosolaniol (NEO) and diacetoxyscirpenol (DAS); zearalenone (ZEN), fumonisin B1 (FB1), fumonisin B2 (FB2) and fusaric acid [10,11,12,13,14,15]. Acute and chronic exposure to these mycotoxins exhibits various toxic effects to plants and animals, and poses a potential health risk for humans [16,17]. Due to the high toxicity and worldwide occurrence of the mycotoxins, maximum levels concerning some major mycotoxins have been set in the European countries [18] and also in China [19]. 

The phase of maize fusariosis with the highest toxicological concern is the ear rot, but large amounts of mycotoxins can also be formed in infected leaves (NIV), rotted stalks (notably ZEN and DON) and whole plants (ZEN) [20]. The variability in the fungal strains is an important issue for food safety, as multiple mycotoxins with different toxicities could be produced. So far, the risks of combined toxicity have been poorly understood, but generally it can be concluded that co-exposure to several different mycotoxins often results in synergistic effects [21]. In addition, the matrix significantly influences the toxin-producing abilities of the mycotoxigenic fungi, leading to complex mycotoxin contamination situations. Therefore, it is a critical issue to investigate the mycotoxin profiles and reveal mycotoxigenic potentials of various *Fusarium* spp. in different substrates.

Several studies have been performed to investigate the relationship between *Fusarium* spp. and mycotoxin production. In Germany, as well as in many other central European countries, *F. graminearum* is the predominant *Fusarium* fungi in wheat followed by *F. culmorum*, both of which have been associated with occurrence of ZEN and DON in wheat and other crops [22,23]. In China, 3-ADON, 15-ADON and NIV are the main mycotoxins produced by *F. graminearum* isolated from wheat ears with clear FHB symptoms [24]. Several other surveys also suggested that *F. solani*, *F. graminearum* and *F. sambucinum* could produce one or more mycotoxins, such as DON in north-central United States [25] and ZEN, NIV, 15-ADON in Argentina [12,26]. However, most of the studies only focused on the main important *Fusarium* fungi isolated from cereal grains, with very little attention paid to other fungal species, such as *Fusarium musae*, *Fusarium fujikuroi*, *Fusarium concentricum*, *Fusarium lateritium*, *Fusarium incarnatum-equiseti*, *Fusarium meridionale* and *Fusarium polyphialidicum*. This study therefore took in account the less studied species for the following reasons: (1) they play an important role in spoilage of grain cereals during storage and marketing; (2) these *Fusarium* species can potentially produce mycotoxins in maize and rice matrices even though they were isolated from other substrates such as banana, green pepper and barley. No previous attempts have been made to study the distributions of all frequently occurring mycotoxins (such as ZEN and its derivatives, type B trichothecenes, type A trichothecenes, FB1, FB2 and fusaric acid), along with some other less studied *Fusarium* metabolites produced by various *Fusarium* spp.

The major focus of this study is to thoroughly investigate the mycotoxin-producing capabilities of twenty *Fusarium* species in different culture substrates. A definitive understanding of the prevalence of *Fusarium* spp. and their associated mycotoxigenic potential is not only critical for the development of strategies for monitoring and managing mycotoxin contamination, but also to obtain a precise picture of the toxicological risks related to maize and rice consumption by humans and animals.

## 2. Results and Discussion

### 2.1. Molecular Characterization of Fusarium Species

**Figure 1:**
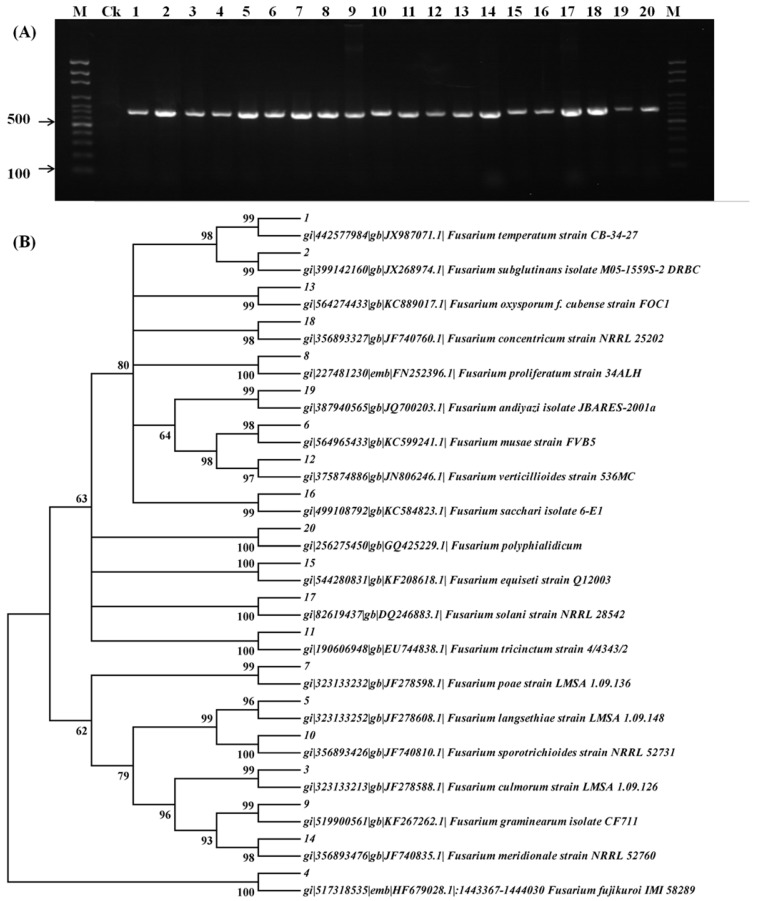
Electrophoresis chromatographs of the *EF-1α* gene from different purified *Fusarium* strains (**A**) and subsequently constructed phylogenetic tree; (**B**). M indicates the 100-bp molecular marker; CK indicates negative control; 1–20 indicates the *Fusarium* strains as described in Table 1.

The electrophoresis chromatograms of the *EF-1α* gene from twenty strains (Table 1) collected from different areas are shown in Figure 1A. The single band observed for all the selected strains demonstrated the purity of the fungi and the species to be *Fusarium* strains.

The phylogenetic tree constructed based on *EF-1α* gene is shown in Figure 1B. After comparison of the targeted gene sequences with the standard sequences in GenBank, the identity of all the *Fusarium* strains was clearly confirmed since the similarities of the sequence between the targeted fungi and the standard one were equal to or above 96%.

### 2.2. Applicability of LC-MS/MS Method

The utilized LC-MS/MS method was established for simultaneous determination of multiple mycotoxins in *Lentinula edodes* in the previous study, and its applicability on PDA, rice and maize was validated. In the present study, the recoveries at concentration levels of 50 μg·kg^−1^ for all mycotoxins spiked into each sample were tested. The experiment was done in quintuplicate. The results showed that satisfactory recoveries with mean values in the range of 72.5%–119.8% in PDA, 72.5%–119.5% in rice and 72.3%–119.6% in maize were obtained for all 33 mycotoxins (Table S1), verifying the suitability of the method employed for determination of the targeted mycotoxins in the above matrices. MRM chromatograms of mycotoxins detected in the media by the selected *Fusarium* species are presented in Figures S1–S4, showing that these mycotoxins can be identified by their retention times and two selective monitoring transitions. 

### 2.3. Principal Component Analysis

Mycotoxin production of *Fusarium* species are influenced greatly by culture conditions [27,28,29]. For instant, too high or low temperature showed inhibition of toxin biosynthesis [28] and thus the appropriate temperature such as 25 °C was adopted in some of related in vitro experiments when investigating multiple *Fusarium* species [30,31]. The temperature effects on mycotoxin production (PDA, 21 days) were initially evaluated between 5 °C and 40 °C in our study, and the results indicated that the sensitivity to temperature varied for different *Fusarium* species. Given that the optimum temperature for majority of the investigated twenty species ranged from 20 °C to 30 °C (results shown in Table S2), 25 °C was chosen and kept constant for the following experiments. The culture medium is also the predominant effect on mycotoxin production by fungi, such as carbohydrate and nitrogen sources [30,32]. Previous reports showed that the carbohydrate-rich media were apparently more favorable for toxin producing [30,33], which was consistent with our results that significantly more mycotoxins were observed in rice and maize media than those in PDA medium (Tables S3–S5), except fusaric acid produced with highest abundance by some of *Fusarium* species on PDA medium. In order to further investigate the mycotoxigenic abilities of various *Fusarium* species on different culture media, PCA was carried out to classify the *Fusarium* strains based on their mycotoxin production. The score plots including PC1 (direction of largest variance) and PC2 (perpendicular to PC1 and against the largest variance) were extracted from the first two principal components, which presented the maximum variability in the data and made it easier to visually discriminate the differences [34]. The value of the loading plot reflects the contribution of each variable to the sample classification in the PCA. The farther from the origin a variable is placed, the higher contribution of that variable made to the PCA model [35]. 

**Figure 2:**
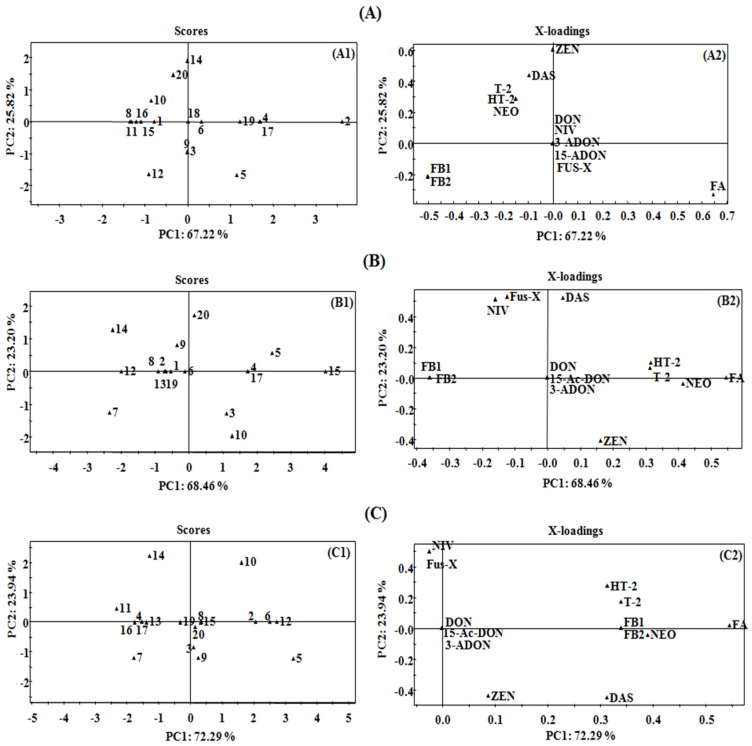
Statistical results of principal component analysis (PCA) of the detected mycotoxins by twenty *Fusarium* species in PDA (**A**), rice (**B**) and maize (**C**) medium. (**A1**), (**B1**) and (**C1**) on the left refer to the score plots showing the locations of the *Fusarium* species; (**A2**), (**B2**) and (**C2**) on the right were designed to the loading plot interpreting the relationships between mycotoxins produced by *Fusarium* species.

In this study, thirteen mycotoxins were detected in the growth media by selected *Fusarium* species and then set as the variables for evaluation. As shown in Figure 2, the first principal component (PC1) and the second principal component (PC2) accounted for 67.22% and 25.82% of the variation for PDA (Figure 2A1), 68.46% and 23.20% for rice medium (Figure 2B1), 72.29% and 23.94% for maize medium (Figure 2C1), respectively. It could be obviously seen that for all the above three culture media the cumulative variance contribution of PC1 and PC2 was more than 90%, proving the significant variability of mycotoxin profiles of different *Fusarium* strains studied.

**Figure 3:**
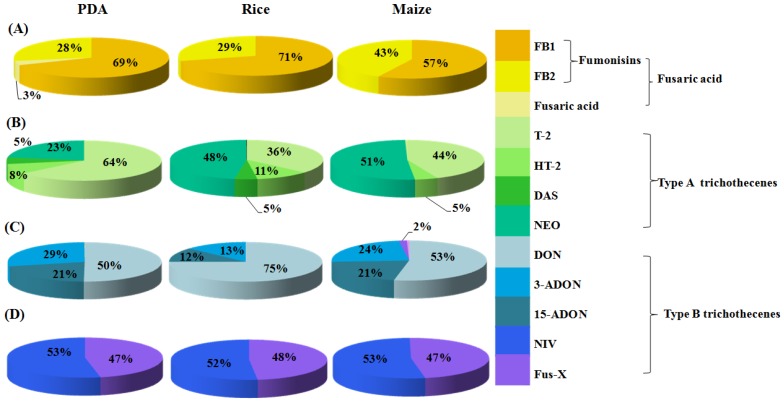
Pie charts of mycotoxin profiling produced by 4 representative *Fusarium* species including *F. proliferatum* (**A**), *F. langsethiae* (**B**), *F. graminearum* (**C**) and *F. meridionale* (**D**) in PDA, rice and maize medium.

For PDA medium (Figure 2A2), fusaric acid correlated positively with PC1 while the fumonisins (FB1 and FB2) correlated negatively, indicating the critical role of these three mycotoxins in the differentiation of the *Fusarium* strains. Type A trichothecenes (T-2 toxin, HT-2 toxin, NEO and DAS) contribute negatively to PC1, but act as significantly positive contributors for PC2, verifying the important role of these four mycotoxins in further discrimination of the *Fusarium* strains. Similarly, the contributors for PC1 for rice medium (Figure 2B2) were fusaric acid, three type A trichothecenes (T-2 toxin, HT-2 toxin and NEO) and fumonisins (FB1 and FB2) while NIV, Fus-X and DAS contributed for PC2. In regard to maize medium, fusaric acid, type A trichothecenes (T-2 toxin, HT-2 toxin, DAS and NEO) and fumonisins (FB1 and FB2) contributed significantly to PC1, and NIV and Fus-X were the contributors for PC2 (Figure 2C2). Based on the mycotoxin profiles in PCA factor loading plots, the detected mycotoxins were divided into three major groups, including the Group I for fusaric acid, which could be subsequently divided into subgroup I for co-occurrence of fusaric acid and fumonisins (FB1 and FB2) and subgroup II for fusaric acid only, Group II for type A trichothecenes (T-2, HT-2, NEO, DAS) and Group III for type B trichothecenes (DON, 15-ADON, 3-ADON, NIV, Fus-X). Consequently, the targeted twenty toxigenic *Fusarium* fungal strains were grouped as shown in Table 2. The mycotoxin profiles of representative strains (*F. proliferatum* (A) for Group I, *F. langsethiae* (B) for Group II, and *F. graminearum* (C) and *F. meridionale* (D) for Group III) in each group cultivated on PDA, maize and rice medium are shown in Figure 3.

### 2.4. Mycotoxin-Producing Capacities of Fusarium Species in Different Growth Media

#### 2.4.1. Group I/Fusaric Acid Producers

Thirteen *Fusarium* strains, including *F. proliferatum*, *F. verticillioides*, *F. fujikuroi*, *F. solani*, *F. temperatum*, *F. subglutinans*, *F. musae*, *F. tricinctum*, *F. oxysporum*, *F. equiseti*, *F. sacchari*, *F. concentricum* and *F. andiyazi*, belonged to Group I due to their fusaric acid producing abilities. Among them, *F. proliferatum*, *F. verticillioides*, *F. fujikuroi* and *F. solani* belonged to subgroup I as producers of both fumonisins and fusaric acid, and the other nine *Fusarium* species were considered to merely produce fusaric acid (Table 2). 

**Figure 4:**
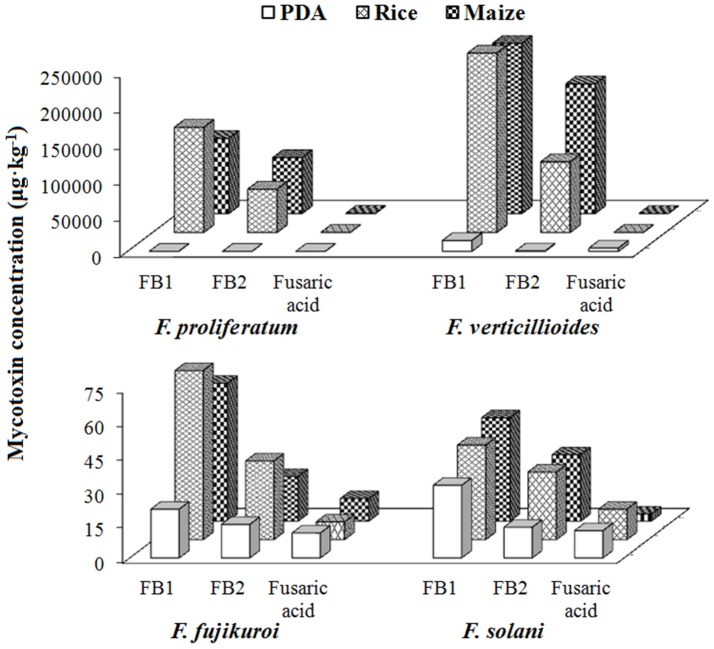
Investigation of the fumonisin B1 (FB1), fumonisin B2 (FB2) and fusaric acid producing abilities of *F. proliferatum*, *F. verticillioides*, *F. fujikuroi* and *F. solani* after incubation for 21 days at 25 °C on PDA, rice and maize media.

*F. proliferatum* and *F. verticillioides* are the major fumonisin producers with average concentrations for FB1 being 10,085 and 15,168 μg·kg^−1^ in PDA, 146,726 and 273,894 μg·kg^−1^ in rice and 104,810 and 237,208 μg·kg^−1^ in maize medium, while the average concentrations for FB2 were 354 and 594 μg·kg^−1^ on PDA, 60,378 and 98,523 μg·kg^−1^ on rice, 77,939 and 180,778 μg·kg^−1^ on maize, respectively (Tables S3–S5 and Figure 4). The high mycotoxin-producing abilities of these two *Fusarium* species found in this study are in good agreement with the previous studies [36,37]. *F. fujikuroi* produced fumonisins as well, but showed much lower concentration levels with values less than 200 μg·kg^−1^ on all the three media, the mycotoxigenic potential of which have been reported to be greatly dependent on the isolated hosts and inoculation conditions [36,38]. Meanwhile, this is the first report about fumonisin production by *F. solani*. 

With regard to the individual mycotoxin, relatively higher contents of FB1 were generated compared to FB2 by the same fungi on the three media (Figure 3A and Figure 4), which have been reported previously [33,39]. The ratios between the two fumonisins (FB1/FB2) for *F. fujikuroi* and *F. solani* were in the range of 1.4–3.1 in all media, but particularly 25.5 and 28.5 in PDA for *F. verticillioides* and *F. proliferatum*, respectively. As expected, the amounts of fumonisins produced in maize and rice were relatively higher than that in PDA, proving the influential role of the composition in different media in fumonisin-producing capabilities of *Fusarium* strains [40,41]. Fusaric acid were also detected with the above four *Fusarium* fungi but the amount was lower than that of fumonisins. 

Nine *Fusarium* strains were found to only produce fusaric acid in our study at average concentration levels ranging from 15 to 12,435 μg·kg^−1^ (Tables S3–S5), and the production levels in PDA were higher than that in rice and maize media especially for *F. subglutinans*, *F. musae*, *F. concentricum* and *F. andiyazi*. In previous studies, *F. sacchari* and *F. andiyazi* were detected to produce low amounts of fumonisin [42,43]. Note that *F. equiseti* was considered as trichothecene producer (DON, 15-ADON, NIV, FUS-X, HT-2 and DAS) [31,44], but it showed a considerable intraspecies variation in profiles of trichothecene production, and even trichothecenes were not observed with some isolates of *F. equiseti* [45]. 

#### 2.4.2. Group II/Type A Trichothecene Producers

Group II was defined as type A trichothecene producer including *F. langsethiae*, *F. sporotrichioides* and *F. polyphialidicum*, which mainly produced one or several of type A trichothecenes, such as T-2, HT-2, NEO and DAS (Table 2).

**Figure 5:**
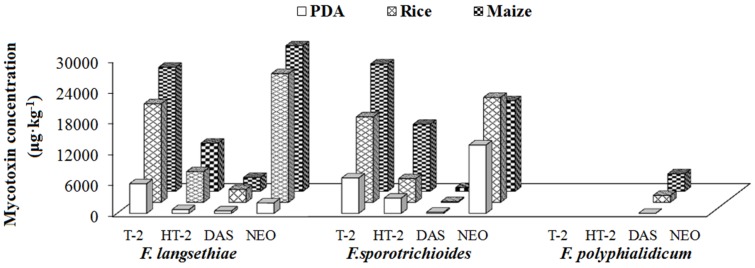
Investigation of the type A trichothecene mycotoxins (T-2, HT-2, NEO and DAS) producing abilities of *F. langsethiae*, *F. sporotrichioides* and *F. polyphialidicum* after incubation for 21 days at 25 °C on PDA, rice and maize media.

Among the Group II *Fusarium* strains, *F. langsethiae* and *F. sporotrichioides* were found to be prolific producers of T-2, which was also demonstrated in Kokkonen et al.’s studies [27,30]. In general, high concentrations of T-2, NEO, and low production of HT-2 and DAS were observed for all the studied substrates (Tables S3–S5, Figure 3B and Figure 5). These results were consistent with the results from Yli-Mattila et al., reporting that *F. langsethiae* and *F. sporotrichioides* produced high levels of T-2 with mean concentrations about 21,700–38,600 μg·kg^−1^, and low mean concentrations of DAS with 90–2800 μg·kg^−1^ [46]. With respect to *F. polyphialidicum*, it appeared to be a rare *Fusarium* species isolated from plant debris collected in South Africa [47], and the mycotoxin producing abilities have been only limitedly investigated up to date, reporting it as FB1 producer [48]. In this study, DAS was found to be produced by this fungus for the first time with mean concentration levels of 23, 1333 and 3386 μg·kg^−1^ in PDA, rice and maize medium, respectively (Tables S3–S5). Additionally, it could be obviously seen that for Group II fungi (Type A trichothecene producers), the highest concentrations of various mycotoxins were produced in maize, followed by rice, and lowest values were observed in PDA.

#### 2.4.3. Group III/Type B Trichothecene Producers

**Figure 6:**
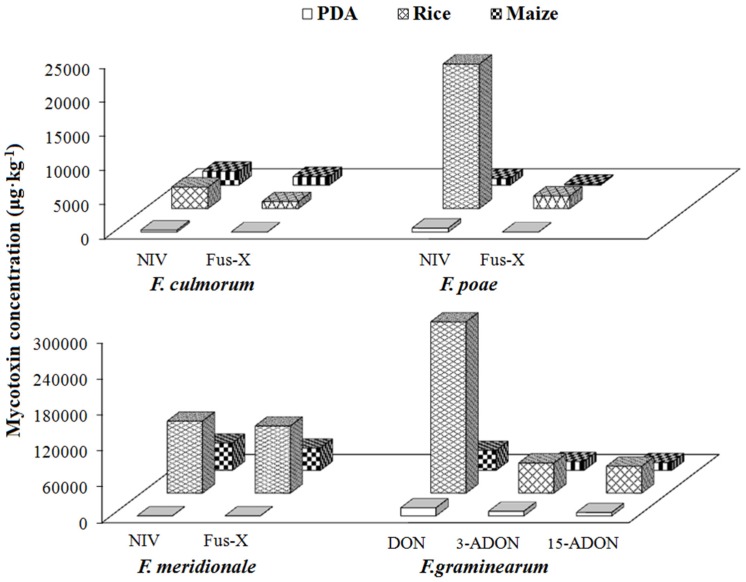
Investigation of the type B trichothecene mycotoxins (NIV, Fus-X, DON, 3-ADON and 15-ADON) producing abilities of *F. culmorum*, *F. poae*, *F. meridionale* and *F. graminearum* after incubation for 21 days at 25 °C on PDA, rice and maize media.

The concentrations of NIV and Fus-X were 269 and 13 μg·kg^−1^ in PDA, 3151 and 1022 μg·kg^−1^ in rice, 2039 and 1260 μg·kg^−1^ in maize produced by *F. culmorum*; 566 and 60 μg·kg^−1^ in PDA, 21,231 and 1838 μg·kg^−1^ in rice, 979 and 137 μg·kg^−1^ in maize produced by *F. poae*; 123 and 107 μg·kg^−1^ in PDA, 120,342 and 112,167 μg·kg^−1^ in rice, 45,453 and 37,175 μg·kg^−1^ in maize produced by *F. meridionale*, respectively (Tables S3–S5, Figure 6). *F. poae* and *F. culmorum* have previously been considered as good producers for NIV and Fus-X [49,50,51]. In the present study, *F. graminearum* produced large amounts of DON, 3-ADON and 15-ADON, with concentration levels in PDA, rice and maize media in the range of 13,532–286,258 μg·kg^−1^, 7700–50,344 μg·kg^−1^ and 5716–44,943 μg·kg^−1^, respectively (Tables S3–S5, Figure 6). In previous studies, two type B trichothecence producing chemotypes were identified, i.e., the NIV and DON chemotypes [52,53,54]. Based on the results of this study, *F. culmorum*, *F. poae* and *F. meridionale* can be grouped into the NIV chemotype, while *F. graminearum* could be classified into the DON chemotype (Table 2).

Another feature with Group III is the co-occurrence of multiple types of mycotoxins in rice and maize media. As consistent with previous in vitro results, ZEN was produced by *F. culmorum* [27,31], *F. meridionale* [1] and *F. graminearum* [27,31] with highest levels herein in rice media. Previous studies indicated that *F. culmorum* showed intraspecies differences in the production of trichothecenes [45]. In our experiments, type A trichothecenes were detected in rice and maize media with *F. culmorum*, as well as *F. poae* and *F. meridionale*, especially significant amount of DAS produced by *F. poae*. 

## 3. Conclusions

Twenty *Fusarium* species isolated from different regions were identified by molecular approaches and then inoculated on three growth media, PDA, rice and maize. The produced mycotoxins were determined quantitatively by LC-MS/MS and results were statistically analyzed using PCA. *Fusarium* species were accordingly divided into three groups, and mycotoxin profiles were thoroughly investigated to provide the direct evidences for clarification of the correlation between different mycotoxigenic fungi, mycotoxins and growth media. The targeted mycotoxin profiling in this study revealed mycotoxigenic potentials of *Fusarium* species in various culture substrates, which would contribute to further research concerning mycotoxin analysis and fungal investigations, as well as provide supporting information for controlling occurrence of fungi and their metabolic mycotoxins from farm to fork to ensure public health safety.

## 4. Materials and Methods

### 4.1. Fungal Strains, Materials and Chemicals

Twenty strains of *Fusarium* fungi were used in this study. *F. temperatum*, *F. subglutinans*, *F. culmorum*, *F. fujikuroi*, *F. langsethiae*, *F. musae*, *F. poae*, *F. proliferatum*, *F. sporotrichioides*, *F. tricinctum* and *F. verticillioides* were provided by Mycothèque de l’Université catholique de Louvain (MUCL, Louvain-la-Neuve, Belgium). The other nine *Fusarium* strains, including *F. graminearum*, *F. oxysporum*, *F. meridionale*, *F. equiseti*, *F. sacchari*, *F. solani*, *F. concentricum*, *F. andiyazi*, and *F. polyphialidicum*, were obtained by single spore isolation in our laboratory. Information about geographical location and plant hosts of all the investigated fungal species are presented in Table 1. Cereal matrices used for preparation of rice and maize medium were purchased from local suppliers, which were all mycotoxins-free as confirmed by liquid chromatography-tandem mass spectrometry (LC-MS/MS).

The mycotoxin standards of aflatoxin B1 (AFB1), aflatoxin B2 (AFB2), aflatoxin G1 (AFG1), aflatoxin G2 (AFG2), aflatoxin M1 (AFM1), aflatoxin M2 (AFM2), HT-2 toxin, T-2 toxin and ochratoxin A (OTA) were supplied by Alexisa (San Diego, CA, USA). 15-ADON and 3-ADON were purchased from Biopure (Tulln, Austria). Fusaric acid, ZEN, zearalanone (ZAN), α-zearalenol (α-ZEL), α-zearalanol (α-ZAL), β-zearalenol (β-ZEL), β-zearalanol (β-ZAL), DON, NIV, deepoxy-DON, sterigmatocystin (SMC), Fus-X, citrinine (CIT), NEO, DAS, mycophenolic acid (MPA), cyclopiazonic acid (CPA), verruculogen (VER), FB1, FB2, patulin (PAT) and gliotoxin were purchased from Sigma-Aldrich (St. Louis, MO, USA). HPLC grade of acetonitrile and methanol were purchased from Merck (Darmstadt, Germany). Other solvents and chemicals were of HPLC or analytical grade from local suppliers. Deionized water purified by Milli-Q water (Millipore, Billerica, MA, USA) was used throughout the experiments.

### 4.2. Molecular Characterization of the Fusarium Strains

*Fusarium* strains were molecularly characterized by examining the sequence of the translation elongation factor 1-alpha (*EF-1α*) gene, known as one of the most pertinent genes for identification of the *Fusarium* species [55]. 

Mycelia plugs from 7-day old potato dextrose agar (PDA) (composition seen in Section 4.3) cultures were transferred to potato dextrose broth (PDB) medium (200 g potato and 20 g glucose per litre) and incubated while shaking (100 rpm) at 28 °C in the dark for 5 days. After incubation, the mycelia were harvested by filtration through filtering cloth, freeze-dried and ground to fine powders using a TissueLyser II system (Qiagen Tissuelyser II, Retsch, Haan, Germany). 

Genomic DNA of strains was extracted based on the Cetyltriethyl Ammnonium Bromide (CTAB) protocol described by Wang et al. [56]. Portions of the *EF-1α* gene were amplified with primers EF1T (3′-ATGGGTAAGGAGGACAAGAC-5′) and EF2T (3′-GGAAGTACCAGTGATCATGTT-5′) in a thermal cycler (T100 Thermal Cycler, Bio-Rad, Foster City, CA, USA). Polymerase chain reaction (PCR) amplification was performed using a modified procedure described [57]. PCR reaction mixtures (total volume of 20 μL) contained 80 ng of fungal genomic DNA template, 1 × PCR buffer (20 mM Tris-HCl pH 8.3, 20 mM KCl, 10 mM (NH_4_)_2_SO_4_, 2 mM MgSO_4_) (TransGen Biotech, Beijing, China), 0.25 mM deoxynucleoside triphosphate (dNTPs) (Dongsheng Biotech, Guangzhou, China), 2.5 U of Easy Taq DNA polymerase (TransGen Biotech, Beijing, China) and 0.2 μM of each primer. The conditions for thermal cycler consisted of an initial denaturation step at 94 °C for 4 min, followed by 30 cycles of denaturation at 94 °C for 30 s, annealing at 56 °C for 40 s and extension at 72 °C for 30 s, then a final extension of 72 °C for 5 min. An aliquot of 8 μL of amplified products was separated by electrophoresis onto a 1% agarose gel, stained with ethidium bromide and photographed under UV light in a Bio-Imaging system (Bio-Rad, Hercules, CA, USA). The incised fragment gels were sent to Invitrogen^TM^ (Shanghai, China) for sequencing. Then the *EF-1α* amplicon sequences (shown in Table S6) generated in this study were compared with sequences available by using the BLAST program [58]. The phylogenetic trees were made using MEGA5.0 for Neighbor-joining (N-J) analysis and the reliability was confirmed by bootstrapping using 1000 random replicates.

### 4.3. Preparation of Different Types of Growth Media

Three typical media including PDA, maize medium and rice medium, were prepared for inoculation and incubation of the *Fusarium* strains. PDA medium (200 g potato, 20 g glucose, and 15–20 g agar per litre) was prepared by autoclaving at 121 °C for 15 min and then 15 mL of molten media was poured into 9 cm diameter sterile Petri dishes. Maize/rice media were prepared by adding 25 mL deionized water into 50 g of mycotoxin-free maize/rice samples, vigorously shaken to prevent clumping, maintained overnight and sterilized in an autoclave for 15 min at 121 °C.

### 4.4. Inoculation of the Targeted Fungal Strains

Prior to the inoculation experiments, each fungal strain was cultured separately on PDA for 7 days at 25 °C for activation of the strain. The inoculation method was conducted as previously described [59,60] with minor modifications. A piece of 6 mm diameter agar disc taken from the margin of a 7-day old colony of each strain grown on PDA was placed in the centre of each test medium and incubated at 25 °C for 21 days. Control samples were prepared following the same procedure without fungal inoculation and each treatment was performed in triplicate. After 21 days of incubation, the media were harvested and dried at 40 °C–50 °C until constant weight was achieved, and then finely ground into homogenous powders and stored in the freezer for mycotoxin analysis. 

### 4.5. Analysis of Multiple Mycotoxins

The mycotoxins produced by various *Fusarium* strains were extracted and simultaneously determined by LC-MS/MS covering a total of thirty three frequently occurring mycotoxins, which has been established in the previous studies [61].

### 4.6. Statistical Analysis

A pie chart and a three-dimensional histogram model were plotted using Microsoft Office Excel 2003 (Microsoft Corp., Redmond, WA, USA). Statistical analysis was performed using SPSS statistical package 17.0 (SPSS Inc., Chicago, IL, USA). One-way analysis of variance (ANOVA) was performed to determine the significance of the main factors and their interactions. *p* < 0.05 was considered statistically significant. Multivariate analysis was used to perform principal component analysis (PCA) by SIMCA-P software 11.0 (Umetrics, Umea, Sweden).

## Figures and Tables

**Table 1:** The information of the *Fusarium* strains used in this study.

Strain No.	Code	*Fusarium* Species	Origin	Host
1	MUCL ^1^ 52463	*Fusarium temperatum*	Belgium	Maize
2	MUCL 43485	*Fusarium subglutinans*	United States	Maize
3	MUCL 42823	*Fusarium culmorum*	Belgium	Wheat
4	MUCL 51036	*Fusarium fujikuroi*	Philippines	Rice
5	MUCL 34988	*Fusarium langsethiae*	-	Wheat
6	MUCL 52574	*Fusarium musae*	Honduras	Banana
7	MUCL 53395	*Fusarium poae*	Belgium	Maize
8	MUCL 43483	*Fusarium proliferatum*	-	-
9	F-1	*Fusarium graminearum*	China	Wheat
10	MUCL 53602	*Fusarium sporotrichioides*	Belgium	Maize
11	MUCL 42821	*Fusarium tricinctum*	Belgium	Wheat
12	MUCL 43478	*Fusarium verticillioides*	United States	Maize
13	B40 = F50/1-i1-B	*Fusarium oxysporum*	China	Barley
14	MC1_30	*Fusarium meridionale*	China	Maize
15	M-12-0203-A1	*Fusarium equiseti*	China	Maize
16	M-12-0501-J1	*Fusarium sacchari*	China	Maize
17	M-12-0601-D12	*Fusarium solani*	China	Maize
18	Q29	*Fusarium concentricum*	China	Green pepper
19	W21	*Fusarium andiyazi*	China	Maize
20	XB4-1	*Fusarium polyphialidicum*	China	Barley

^1^ MUCL Mycothèque de l’Université catholique de Louvain (Louvain-la-Neuve, Belgium).

**Table 2:** Grouping of mycotoxigenic *Fusarium* species using mycotoxin profiles.

Group		Strain No.	*Fusarium* Species	Major Mycotoxins Produced	Other Mycotoxins Produced
Group I (Fusaric acid)	Subgroup I (Fumonisins and fusaric acid)	8	*F. proliferatum*	FB1, FB2, Fusaric acid	-
		12	*F. verticillioides*	FB1, FB2, Fusaric acid	-
		4	*F. fujikuroi*	FB1, FB2, Fusaric acid	-
		17	*F. solani*	FB1, FB2, Fusaric acid	-
	Subgroup II (Fusaric acid only)	1	*F. temperatum*	Fusaric acid	-
		2	*F. subglutinans*	Fusaric acid	-
		6	*F. musae*	Fusaric acid	-
		11	*F. tricinctum*	Fusaric acid	-
		13	*F. oxysporum*	Fusaric acid	-
		15	*F. equiseti*	Fusaric acid	-
		16	*F. sacchari*	Fusaric acid	-
		18	*F. concentricum*	Fusaric acid	-
		19	*F. andiyazi*	Fusaric acid	-
Group II (Type A trichothecenes)		5	*F. langsethiae*	T-2, HT-2, NEO, DAS	-
		10	*F. sporotrichioides*	T-2, HT-2, NEO, DAS	-
		20	*F. polyphialidicum*	DAS	-
Group III (Type B trichothecenes)		3	*F. culmorum*	NIV, Fus-X	T-2, HT-2, NEO, ZEN
		7	*F. poae*	NIV, Fus-X	T-2, HT-2, NEO, DAS
		14	*F. meridionale*	NIV, Fus-X	NEO, ZEN
		9	*F. graminearum*	DON, 15-ADON, 3-ADON	ZEN

## Supplementary Materials

The following are available online at www.mdpi.com/2072-6651/9/1/6/s1, Figure S1: LC-MS/MS chromatogram of FB1, FB2 and fusaric acid ((**a**): standards; (**b**): samples: rice medium, *F. proliferatum*), Figure S2: LC-MS/MS chromatogram of four type A trichothecenes ((**a**): standards; (**b**): samples: rice medium, *F. langsethiae*), Figure S3: LC-MS/MS chromatogram of five type B trichothecenes ((**a**): standards; (**b**): samples: NIV, Fus-X: maize medium, *F. poae*; DON, ADONs: maize medium, *F. graminearum*), Figure S4: LC-MS/MS chromatogram of ZEN ((**a**): standards; (**b**): samples: rice medium, *F. graminearum*), Table S1: Relative recoveries of the thirty three targeted mycotoxins at concentration of 50 μg·kg^−1^ spiked in PDA, rice and maize samples using ^13^C-AFB1, ^13^C-OTA, ^13^C-T-2, ^13^C-DON and ^13^C-ZEN as the internal standards (*n* = 5), Table S2: Optimum temperatures for mycotoxin production of *Fusarium* species (PDA, 21 days), Table S3: Mycotoxins profiles of 20 species of toxicogenic *Fusarium* spp. in PDA medium under culture condition of 25 °C for 21 days (µg·kg^−1^), Table S4: Mycotoxins profiles of 20 species of toxicogenic *Fusarium* spp. in rice medium under culture condition of 25 °C for 21 days (µg·kg^−1^), Table S5: Mycotoxins profiles of 20 species of toxicogenic *Fusarium* spp. in maize medium under culture condition of 25 °C for 21 days (µg·kg^−1^), Table S6: *EF-1α* Sequences of 20 *Fusarium* species.
