# Supplementary material for: Mycotoxigenic Potentials of Fusarium Species in Various Culture Matrices Revealed by Mycotoxin Profiling
Source: Toxins (Basel). 2016 Dec 26;9(1):6. doi: 10.3390/toxins9010006 (PMC5308239; doi:10.3390/toxins9010006)
Supplement: Supplementary file 1 [file toxins-09-00006-s001.pdf]

Supplementary Materials: Mycotoxigenic Potentials of *Fusarium* Species in Various Culture Matrices Revealed by Mycotoxin Profiling

Wen Shi, Yanglan Tan, Shuangxia Wang, Donald M. Gardiner, Sarah De Saeger, Yucai Liao, Cheng Wang, Yingying Fan, Zhouping Wang and Aibo Wu

Table S1. Relative recoveries of the thirty three targeted mycotoxins at concentration of 50 μg·kg^{-1} spiked in PDA, rice and maize samples using ^{13}C-AFB1, ^{13}C-OTA, ^{13}C-T-2, ^{13}C-DON and ^{13}C-ZEN as the internal standards (n = 5).

Mycotoxins	PDA	Rice	Maize
AFB1	108.8 ± 16.4	119.5 ± 6.1	102.0 ± 5.8
AFB2	119.8 ± 8.4	110.7 ± 5.1	88.4 ± 0.7
AFG1	106.4 ± 13.1	107.8 ± 8.3	94.1 ± 10.2
AFG2	112.3 ± 7.4	97.1 ± 3.3	81.6 ± 0.4
AFM1	89.6 ± 9.7	87.7 ± 5.5	90.3 ± 4.4
AFM2	114.4 ± 7.7	112.9 ± 4.8	116.2 ± 0.4
SMC	108.1 ± 8.5	102.4 ± 5.8	100.1 ± 17.3
NEO	101.9 ± 8.6	97.3 ± 16.5	96.0 ± 11.0
OTA	111.3 ± 6.9	96.3 ± 18.5	95.8 ± 6.5
DAS	90.7 ± 12.2	94.6 ± 6.6	110.9 ± 9.0
T-2	95.7 ± 17.3	112.6 ± 5.5	72.3 ± 4.0
HT2	91.6 ± 13.2	103.6 ± 6.8	103.2 ± 19.6
MPA	114.5 ± 14.7	115.2 ± 8.1	119.6 ± 13.6
FB1	89.8 ± 10.3	106.7 ± 3.9	99.1 ± 11.1
FB2	97.2 ± 6.1	86.5 ± 3.6	85.0 ± 12.4
VER	79.6 ± 5.3	88.2 ± 3.4	95.9 ± 2.1
DON	83.6 ± 16.4	101.3 ± 19.2	107.9 ± 16.0
Fus X	99.3 ± 15.4	98.2 ± 12.3	85.8 ± 7.6
Deep-DON	106.9 ± 18.7	91.6 ± 8.1	116.5 ± 6.2
15-DON	101.2 ± 13.3	102.5 ± 9.1	101.0 ± 20.5
3-ADON	90.0 ± 14.3	90.3 ± 13.5	103.8 ± 19.5
CPA	111.8 ± 16.0	107.8 ± 12.4	86.6 ± 12.1
ZEN	107.1 ± 6.9	75.2 ± 4.0	89.6 ± 13.8
ZAN	110.3 ± 6.9	102.5 ± 16.1	110.3 ± 14.9
α-ZOL	83.6 ± 9.6	103.5 ± 6.9	101.7 ± 7.9
α-ZAL	108.9 ± 8.5	98.9 ± 8.4	86.1 ± 11.4
β-ZOL	72.5 ± 3.4	93.3 ± 2.5	81.7 ± 5.3
β-ZAL	92.1 ± 3.5	88.3 ± 4.4	87.1 ± 5.5
Fusaric acid	102.1 ± 11.2	119.4 ± 2.8	89.5 ± 9.9
Glitoxin	108.8 ± 14.5	96.9 ± 13.2	92.8 ± 10.9
CIT	99.3 ± 16.0	115.9 ± 2.3	107.3 ± 10.6
PAT	113.8 ± 3.5	72.5 ± 4.1	100.5 ± 15.5
NIV	81.3 ± 7.9	111.0 ± 9.5	104.4 ± 13.2

Table S2. Optimum temperatures for mycotoxin production of *Fusarium* species (PDA, 21 days).

Strain No.	*Fusarium* Species	Optimum Temperature for Mycotoxin Production (°C)
1	*Fusarium temperatum*	20
2	*Fusarium subglutinans*	20
3	*Fusarium culmorum*	20
4	*Fusarium fujikuroi*	25
5	*Fusarium langsethiae*	15
6	*Fusarium musae*	25
7	*Fusarium poae*	25
8	*Fusarium proliferatum*	30
9	*Fusarium graminearum*	30
10	*Fusarium sporotrichioides*	15
11	*Fusarium tricinctum*	30
12	*Fusarium verticillioides*	30
13	*Fusarium oxysporum*	35
14	*Fusarium meridionale*	30
15	*Fusarium equiseti*	25
16	*Fusarium sacchari*	25
17	*Fusarium solani*	25
18	*Fusarium concentricum*	30
19	*Fusarium andiyazi*	30
20	*Fusarium polyphialidicum*	25

Table S3. Mycotoxins profiles of 20 species of toxicogenic *Fusarium* spp. in PDA medium under culture condition of 25 °C for 21 days (μg·kg^{-1}).

**Strain No.**	**Type B Trichothecenes**					**Type A Trichothecenes**				**Fumonisins**		**Fusaric Acid**	**ZEN**
	**DON**	**3-ADON**	**15-ADON**	**NIV**	**FUS-X**	**T-2**	**HT-2**	**DAS**	**NEO**	**FB1**	**FB2**		
1												1469 ± 237	
2												12,435 ± 667	
3				269 ± 15	13 ± 1							4 ± 0.3	4.4 ± 0.4
4										21 ± 4	15 ± 0.8	11 ± 0.9	
5						5789 ± 630	737 ± 94	482 ± 73	2030 ± 379				
6												4232 ± 175	
7				566 ± 36	60 ± 6								
8										10,085 ± 883	354 ± 66	34 ± 3	
9	13,532 ± 486	7700 ± 432	5716 ± 714									5 ± 0.3	5.2 ± 0.5
10						6904 ± 902	2967 ± 409	231 ± 50	13,334 ± 693				
11												146 ± 11	
12										15,168 ± 1227	594 ± 77	4642 ± 500	
13													
14				123 ± 31	107 ± 6								41 ± 6
15												698 ± 335	
16												408 ± 51	
17										32 ± 4	14 ± 3	12 ± 0.7	
18												3410 ± 228	
19												6464 ± 379	
20								23 ± 5					

Table S4. Mycotoxins profiles of 20 species of toxicogenic *Fusarium* spp. in rice medium under culture condition of 25 °C for 21 days (μg·kg^{-1}).

Strain No.	Type B Trichothecenes					Type A Trichothecenes				Fumonisins		Fusaric Acid	ZEN
	DON	3-ADON	15-ADON	NIV	FUS-X	T-2	HT-2	DAS	NEO	FB1	FB2		
1												344 ± 47	
2												34 ± 13	
3				3151 ± 205	1022 ± 98								817 ± 37
4										74 ± 16	35 ± 7	7.8 ± 0.6	
5						19,228 ± 3783	5983 ± 231	2507 ± 93	25,079 ± 465				
6												938 ± 103	
7				21,231 ± 1012	1838 ± 69	140 ± 29	25 ± 0.9	1274 ± 257	213 ± 55				
8										146,726 ± 12746	60,378 ± 2376	36 ± 7	
9	286,258 ± 8794	50,344 ± 574	44,943 ± 868										297 ± 67
10						16,651 ± 3055	4623 ± 568	244 ± 19	20,456 ± 493				
11													
12										273,894 ± 3464	98,523 ± 10,352	110 ± 9	
13												77 ± 11	
14				120,342 ± 4967	112,167 ± 13,653				436 ± 57				185 ± 32
15												828 ± 48	
16													
17										42 ± 6	30 ± 6	13 ± 1	
18													
19												19 ± 2	
20								1333 ± 90					

Table S5. Mycotoxins profiles of 20 species of toxicogenic *Fusarium* spp. in maize medium under culture condition of 25 °C for 21 days ($\mu g \cdot kg^{-1}$).

Strain No.	Type B Trichothecenes					Type A Trichothecenes				Fumonisins		Fusaric Acid	ZEN
	DON	3-ADON	15-ADON	NIV	FUS-X	T-2	HT-2	DAS	NEO	FB1	FB2		
1													
2												657 ± 70	
3				2039 ± 345	1260 ± 74	74 ± 11	13 ± 1		33 ± 6				204 ± 32
4										61 ± 17	20 ± 3	10 ± 2	
5						24,179 ± 2013	9376 ± 433	2668 ± 105	28,365 ± 1022				
6												733 ± 23	
7				979 ± 79	137 ± 36	8.1 ± 0.7		1102 ± 95	61 ± 18				
8										104,810 ± 8563	77,939 ± 6036	344 ± 62	
9	33,236 ± 2584	14,920 ± 2468	13,038 ± 1693		1060 ± 74								91 ± 9
10						24,836 ± 9005	13,053 ± 768	712 ± 12	17,656 ± 1245				
11												28 ± 4	
12										237,208 ± 8654	180,778 ± 6438		
13												80 ± 9	
14				45,453 ± 4049	37,175 ± 3529				126 ± 5				79 ± 15
15												368 ± 31	
16												15 ± 0.8	
17										146 ± 10	49 ± 8	3.2 ± 0.1	
18													
19												258 ± 32	
20								3386 ± 103					

Table S6. *EF-1α* Sequences of 20 *Fusarium* species.

Strain No. 1
GCACGTCGACTCTGGCAAGTCGACCACTGTGAGTACTACCCTCGACGATGAGCTTATCTG<br>CCATCGTTATCCCGACCAAGACCTGGCGGGCGTCTCAAAAGATAACATGCTGACATCGC<br>TTCACAGACCGGTCACTTGATCTACCAGTGCGGTGGTATCGACAAGCGAACCATCGAGA<br>AGTTCGAGAAGGTTAGTTACTTTCCCTTCGATCGCGCGTCCTTTGCCCATCGATTCTCCAT<br>ACGACTCGAAACGTGCCCGCTACCCCGCTCGAGACCAAAAAATTTGCGATATGACCGTA<br>ATTTTTTTGGTGGGGCATTTACCCCGCCACTCGAGCAATGCGCGTTTCTGCCCTCCCATTG<br>CCACAACCTTCTGAGCGCATCGTCACGTGTTAAGCAGTCACTAACCATTCGTCAATAGGA<br>AGCCGCTGAGCTCGGTAAGGGTTCCTTCAAGTACGCCTGGGTTCTTGACAAGCTCAAGGC<br>CGAGCGTGAGCGTGGTATCACCATCGATATTGCTCTCTGGAAGTTCGAGACTCCTCGCTA<br>CTATGTCACTGTCATTGGTATGTTGTCGCTCATGCCTCGTTCTCCCTTAATTCGTACTAACA<br>TATCACTCAGACGCTCCCGGTCATCGTGACTTCATCAAGAACATGATCCGGGGTGAATTT<br>CCAA
Strain No. 2
CGACTCTGGCAGTCGACCACTGTGAGTACTACCCTCGACGATGAGCTTATCTGCCATCGT<br>TATCCCGACCAAGACCTGGCGGAGTATCTCAAAGATAACATGCTGACATCGCTCCACAG<br>ACCGGTCACTTGATCTACCAGTGCGGTGGTATCGACAAGCGAACCATCGAGAAGTTCGA<br>GAAGGTTAGTTACTTTCCCTTCGATCGCGCGTCCTTTGCCCATCGATTCTCCATTCGACTC<br>GAAACGTGCCCGCTACCCCGCTCGAGACCAAAAATTTTGCGATATGACCGTAATTTTTTT<br>TGGTGGGGCATTTACCCCGCCACTCGAGCGATGCGCGTTTCTGCCCTCTCATTTTCACAAC<br>CTTCTGAGCGCATCGTCACGTGCTAAGCAGTCACTAACCATTCAACAATAGGAAGCCGC<br>CGAGCTCGGTAAGGGTTCCTTCAAGTACGCCTGGGTTCTTGACAAGCTCAAGGCTGAGCG<br>TGAGCGTGGTATCACCATCGATATTGCTCTCTGGAAGTTCGAGACTCCTCGCTACTATGTC<br>ACCGTCATTGGTATGTTGTCGCTCATGCATTGTTCTCCCTTTATTCGTACTAACATATCACT<br>CAGACGCTCCCGGTCATCGTGACTTCATCAAGAACATGATCCGGGGCTTTTCTCCAAAA
Strain No. 3
GATCGGCCACGTCGACTCTGGCAAGTCGACCACTGTGAGTACCACTGCATCCCAACCCC<br>GCCGATACTTGGCGGGGTAGTTTCAAATTTCCAATGTGCTGACATACTTTGATAGACCGG<br>TCACTTGATCTACCAGTGCGGTGGTATCGACAAGCGAACCATCGAGAAGTTCGAGAAGG<br>TTGGTCTCATTTTCCTCGATCGCGCGCCCTTTTCCCTTTCGAAACATCATTCGAATCGCCCT<br>CACACGACGACTCGATACGCGCCTGTTACCCCGCTCGAGGTCAAAAATTTTGCGGCTTTG<br>TCGTAATTTTTCTGGTGGGGCTCATACCCCGCCACTCGAGCGACAGGCGCTTGCCCTCTTC<br>CCACAAACCATTCCCTAGGCGCGCACCATCACGTGTCAATCAGTTACTAACCACCTGTCA<br>ATAGGAAGCCGCCGAGCTCGGTAAGGGTTCCTTCAAGTACGCCTGGGTTCTTGACAAGCT<br>CAAAGCCGAGCGTGAGCGTGGTATCACCATTGATATCGCTCTCTGGAAGTTCGAGACTCC<br>TCGCTACTATGTCACCGTCATTGGTATGTTGTCACTACTGCTGTCATCACATTCTCATACTA<br>ACACGACTATCAGACGCTCCCGGTCACCGTGATTTCATCAAGAACATGATCACTGGTACT<br>TCC
Strain No. 4
GATCGGCCACGTCGACTCTGGCAAGTCGACCACTGTGAGTACTACCCTCGACGATGAGC<br>TTATCTGTCATCGTGATCCTGACCAAGATCTGGCGGGGTATATCTCAGAAGACAATATGC<br>TGACATCGCTTCACAGACCGGTCACTTGATCTACCAGTGCGGTGGTATCGACAAGCGAAC<br>CATCGAGAAGTTCGAGAAGGTTAGTCACTTTCCCTTCGATCGCGCGTCCTTTGCCCACCG<br>ATTTCCCTTACGATTCGAAACGTGCCTGCTACCCCGCTCGAGACCAAAAATTTTGCGATA<br>TGACCGTAATTTTTTTTGGTGGGGCATTTACCCCGCCACTCGAGTGATGGGCGCGTTTTGC<br>CCTTTCCTGTCCACAACCTCAATGAGCGCATTGTCACGTGTCAAACTAACCATTCGACAA<br>TAGGAAGCCGCTGAGCTCGGTAAGGGTTCCTTCAAGTACGCCTGGGTTCTTGACAAGCTC<br>AAGGCCGAGCGTGAGCGTGGTATCACCATCGATATTGCTCTCTGGAAGTTCGAGACTCCT<br>CGCTACTATGTCACCGTCATTGGTATGTTGTCGCTCATGCGTCATTCTACTTATTCATACTA

ACATATCATTCAGACGCTCCCGGTCACCGTGATTTCATCAAGAACATGATCACTGGTACT
TCCA

Strain No. 5
AGACATAGTCTAACACTGTCAGTACCTCTGCATCCCAACCCCGCCGACAATTGGCGGGGT
AGTTTCAAACTTCCTCTTTGCTGACATGCTTTGACAGACCGGTCACTTGATCTACCAGTGC
GGTGGTATCGACAAGCGAACCATCGAGAAGTTCGAGAAGGTTGGTCTCATTTTCCTCGAT
CGCGCGCCCTTCTTCCCATCGATCCATCATTCAAATCGCTCTCATACAACAACTCGACAA
GCGCCGGTTACCCCGCTCGAGTTTAAAATTTTACGGCCGTGTCGTAATTTTTTTGTGGTG
GGGCTCATACCCCGCCACTCGAGTGACAGGGGCTCTTCCTTCCCACATAGCCATTTACAT
GGGCGCGCATCATCACGTGTCAATCAGTCACTAACCACCTGTCAATAGGAAGCCGCTGA
GCTCGGTAAGGGTTCCTTCAAGTACGCCTGGGTTCTTGACAAGCTCAAAGCCGAGCGTGA
GCGTGGTATCACCATTGATATCGCTCTCTGGAAGTTCGAGACTCCTCGCTACTATGTCACC
GTCATTGGTATGTTATCATTATTGTCTTCATCACATTATCATACTAACATGCCTGCCAGAC
GCTCCCGGGCACCGTGATTTCATCAAGAACATGATCACTGGGTACTTCCCAAAAAA

Strain No. 6
CTATCGGCCACGTCGACTCTGGCAAGTCGACCACTGTGAGTACTACCCTTGACGATGAGC
TTATCGGCCATCGTAAACCCGGCCAAGACCTGGCAGGGTATTTTTCAAAGAAAACATGCT
GACATCGCTTCACAGACCGGTCACTTGATCTACCAGTGCGGTGGTATCGACAAGCGAAC
CATCGAGAAGTTCGAGAAGGTTAGTCACTTTTCCTTCTATCGCGCGTTCTTTGCCCATCGA
TTTCCCCCTACGACTCGAAACGTACCCGCTACCCCGCTCGAGCCCAAAAATTTTGCGATA
TGACCGTAATTTTTTCTGGTGGAGCATTTACCCCGCCACTCGAGCGGCGCGTTTCTGCCCT
CTCCCATTCCACAACCTCACTGAGCTCATCGTCACGTGTCAAGCAGTCACTAACCATCCG
ACAATAGGAAGCCGCTGAGCTCGGTAAGGGTTCCTTCAAGTACGCCTGGGTTCTTGACA
AGCTCAAGGCCGAGCGTGAGCGTGGTATCACCATCGATATCGCTCTCTGGAAGTTCGAG
ACTCCTCGCTACTATGTCACCGTCATTGGTATGTCGTCGCTCTTACTCCGTTCTATATCTCC
TATTACTAACACATCACATAGACGCTCCCGGTCACCGTGATTTCATCAAGAACATGATCA
CTGG

Strain No. 7
AGTCGAACTCTGGCAAGTCGACCACTGTAAGTACAACCGAATCTCAACTCCGCTTTGACA
TGGCGGGGTAGACTCAACATGCACTTGTGCTAACATGCTTGACAGACCGGTCACTTGATC
TACCAGTGCGGTGGTATCGACAAGCGAACCATCGAGAAGTTCGAGAAGGTTGGTCTCAT
TTTCCTCGATCGCGCGCCCTACTTTCCCTCGATCCATCACTCGAATCGCTCTTATACGACT
CGACACACACCTGTTACCCCGCTCGAGTCCGAATTTTTACGATTTTGTCGTAAAAATTTTC
GGTGGGGCTTATACCCCGCCACTCGAGCGATTGCATTTCTTTGGGCGCGAATCGTCACGT
GTCAATCTGTTACTAACCACCTGTCAATAGGAAGCCGCCGAGCTCGGTAAGGGTTCTTTC
AAGTACGCCTGGGTTCTTGACAAGCTCAAGGCCGAGCGTGAGCGTGGTATCACCATCGA
TATCGCTCTCTGGAAGTTCGAGACTCCCCGCTACTATGTCACCGTCATTGGTATGTTGTCA
CTACTACCTCAGTCACTCACATTCTCATACTAACTCATCTATCAGACGCTCCCGGTCACCG
TGATTTCATCAAGAACATGATCATGGGGTACTTCCAA

Strain No. 8
TGACAGTCGACTCTGGCAAGTCGACCACTGTGAGTACTACCCTGGACGTTGAGCTTATCT
GCCATCGTGATCCTGACCAAGATCTGGCGGGGTACATCTTGGAAGACAACATGCTGACA
TCGCTTCACAGACCGGTCACTTGATCTACCAGTGCGGTGGTATCGACAAGCGAACCATCG
AGAAGTTCGAGAAGGTTAGTCACTTTCCCTTCGATCGCGCGTCCTCTGCCCACCGATTTC
ACTTGCGATTCGAAACGTGCCTGCTACCCCGCTCGAGACCAAAAATTTTGCGATATGACC
GTAATTTTTTTGGTGGGGCATTTACCCCGCCACTCGAGCGATGGGCGCGGTTTTGCCCTTT
CCTGTCCACAACCTCAATGAGCGCATTGTCACGTGTCAAGCAGCGACTAACCATTCGAC
AATAGGAAGCCGCTGAGCTCGGTAAGGGTTCCTTCAAGTACGCCTGGGTTCTTGACAAGC
TCAAGGCCGAGCGTGAGCGTGGTATCACCATCGATATTGCTCTCTGGAAGTTCGAGACTC
CTCGCTACTATGTCACCGTCATTGGTATGTTGTCGCTCATACCTCATCCTACTTCCTCATAC
TAACACATCATTCAGACGCTCCCGGTCACCGTGATTTCATCAAGAACATGATCATGGGTA
TTTCC

Strain No. 9
AGTCGAACTCTGGCAAGTCGACCACTGTGAGTACCACCGCATCCCAACCCCGCCGACAC
TTGGCGGGGTAGTTTCAAATTTCCAATGTGCTGACATACTTTGATAGACCGGTCACTTGAT
CTACCAGTGCGGTGGTATCGACAAGCGAACCATCGAGAAGTTCGAGAAGGTTGGTCTCA
TTTTCCTCGATCGCGCGCCCTTTTCCTTTCGAAATATCATTCGAATCGCACTCACACGACG
ACTCGATACGCGCCTGTTACCCCGCTCGAGGTCAAAAATTTTGCGGCTTTGTCGTAATTTT
TTTCCCTGGTGGGGCTCATACCCCGCCACTCGAGCGACAGGCGTTTGCCCTCTTCCCACA
AACCATTCCCTGGGCGCTCATCATCACGTGTCAACCAGTCACTAACCACCTGTCAATAGG
AAGCCGCCGAGCTCGGTAAGGGTTCCTTCAAGTACGCCTGGGTTCTTGACAAGCTCAAA
GCCGAGCGTGAGCGTGGTATCACCATTGATATCGCCCTCTGGAAGTTCGAGACTCCTCGC
TACTATGTCACCGTCATTGGTATGTTGTCACCACTGCTGTCATCACATTCTCATACTAACA
TGGCTATCAGACGCTCCCGGTCACCGTGATTTCATCAAGAACATGATCATGGGTTACCTT
TA

Strain No. 10
AGTCGACTCTGGCAAGTCGACCACTGTGAGTACATCTGCATCACAACCCCGCCCAGACTT
GGCGGGGTAGTTTCAATCATCATTTTTACTGACATGCTTTGACAGACCGGTCACTTGATCT
ACCAGTGCGGTGGTATCGACAAGCGAACCATCGAGAAGTTCGAGAAGGTTGGTCTCATT
TTCCTCGATCGCGCGCCCTTCTTCCCATCGACCCATCATTCGAATCGCTCTCATACGACGA
CTCGACAAGCGCCTGTTACCCCGCTCGAGTTCAAAAATTTTACGGCTGTGTCGTGATTTTT
TGATAGTGGGGCTCATACCCCGCCGCTCGAGTGACAGGCGCTTTTGCCCTTCCCACACAT
CCATTTACATGGGCGCGCATCATCACGTGTCAATCAGTCACTAACCACCTGTCAATAGGA
AGCCGCCGAGCTCGGTAAGGGTTCCTTCAAGTACGCCTGGGTTCTTGACAAGCTCAAAGC
CGAGCGTGAGCGTGGTATCACCATTGATATCGCTCTCTGGAAGTTCGAGACTCCTCGCTA
CTATGTCACCGTCATTGGTATGTTGTCACTATTGCCTTCATCACATTCTCATACTAACATG
CCTACCAGACGCTCCCGGTCACCGTGATTTCATCAAGAACATGATCATGGGGACTTTTCC

Strain No. 11
CTGTATCGGCCACGTCGACTCTGGCAAGTCGACCACTGTAAGTACAACCATCAGCGGGTT
GCTTATATGCACTCGGAATCCGCCAAACCTGGCGGGGTATCACCACAACATTTTGCTAAC
TTTTGACAGACCGGTCACTTGATCTACCAGTGCGGTGGTATCGACAAGCGAACCATCGAG
AAGTTCGAGAAGGTTAGTCAATATCCCTTCGATTACGCGCGCTCCCATCGATTCCCACGA
TTCGCTCCCTCACTCGAAACGCATCCATTACCCCGCTCGAGCCCGAGAATTTTGCGGTGC
GACCGTGATTTTTTTCTGGTGGGGTATCTTACCCCGCCACTCGAGTGACGGATGCGCTTGC
CCTGTTCCCACAAAACCTTACCACCCTGTCGCGCACTACATGTCTTGCAGTCACTAACCA
CTGGACAATAGGAAGCCGCCGAGCTCGGAAAGGGTTCCTTCAAGTACGCCTGGGTTCTT
GACAAGCTCAAAGCCGAGCGTGAGCGTGGTATCACCATTGATATCGCTCTCTGGAAGTTC
GAGACTCCTCGCTACTATGTCACCGTCATTGGTATGTTGTCACTGTCTCACACTATCATGC
TTGCATCATGCTAACATCTCTGTCAGATGCCCCCGGTCATCGTGATTTCATCAAGAACAT
GATCACTGGTACTTCC

Strain No. 12
ACTCGGCCACGTCGACTCTGGCAAGTCGACCACTGTGAGTACTACCCTTGACGATGAGCT
TATCGGCCATCGTAAACCCGGCCAAGACCTGGCGGGGGATTTCTCAAAGAAAACATGCT
GACATCGCTTCACAGACCGGTCACTTGATCTACCAGTGCGGTGGTATCGACAAGCGAAC
CATCGAGAAGTTCGAGAAGGTTAGTCACTTTTCCTTCTATCGCGCGTTCTTTGCCCATCGA
TTCCCCCCTACGACTCGAAACGTACCCGCTACCCCGCTCGAGCCCAAAAATTTTGCGATA
CGACCGTAATTTTTTCTGGTGGGGCATTTACCCCGCCACTCGAGCGGCGCGTTTCTGCCCT
CTCCCATTCCACAACCTCACTGAGCTCATCGTCACGTGTCAAGCAGTCACTAACCATCCG
ACAATAGGAAGCCGCTGAGCTCGGTAAGGGTTCCTTCAAGTACGCCTGGGTTCTTGACA
AGCTCAAGGCCGAGCGTGAGCGTGGTATCACCATCGATATCGCTCTCTGGAAGTTCGAG
ACTCCTCGCTACTATGTCACCGTCATTGGTATGTTGTCGCTCTTATTCCGTTCTATATCTTC
TATTACTAACACATCACATAGACGCTCCCGGTCACCGTGATTTCATCAAGAACATGATCA
CTGGTACTTCC

Strain No. 13

CGACTCTGGCAAGTCGACCACTGTGAGTACTCTCCTCGACAATGAGCATATCTGCCATCG
TCAATCCCGACCAAGACCTGGCGGGGTATTTCTCAAAGTCAACATACTGACATCGTTTCA
CAGACCGGTCACTTGATCTACCAGTGCGGTGGTATCGACAAGCGAACCATCGAGAAGTT
CGAGAAGGTTAGTCACTTTCCCTTCAATCGCGCGTCCTTTGCCCATCGATTTCCCCTACGA
CTCGAAACGTGCCCGCTACCCCGCTCGAGACCAAAAATTTTGCAATATGACCGTAATTTT
TTTTGGTGGGGCACTTACCCCGCCACTTGAGCGACGGGAGCGTTTGCCCTCTTACCATTCT
CACAACCTCAATGAGTGCGTCGTCACGTGTCAAGCAGTCACTAACCATTCAACAATAGG
AAGCCGCTGAGCTCGGTAAGGGTTCCTTCAAGTACGCCTGGGTTCTTGACAAGCTCAAGG
CCGAGCGTGAGCGTGGTATCACCATCGATATTGCTCTCTGGAAGTTCGAGACTCCTCGCT
ACTATGTCACCGTCATTGGTATGTTGTCGCTCATGCTTCATTCTACTTCTCTTCGTACTAAC
ATATCACTCAGACGCTCCCGGTCACCGTGATTTCATCAAGAACATGACC

Strain No. 14

GCAACGTCGACTCTGGCAAGTCGACCACTGTGAGTACCACCACATCCCAACCCCGCCGA
CACTTGGCGGGGTAGTTTCAAATTTCCAACGTGCTGACATACTTTGATAGACCGGTCACT
TGATCTACCAGTGCGGTGGTATCGACAAGCGAACCATCGAGAAGTTCGAGAAGGTTGGT
CTCATTTTCCTCGATCGCGCGCCTTTTTCCCTTTCGAAATATCATTCGAATCGCCCTCACAC
GACGACTCGATACGCGCCTGTTACCCCGCTCGAGGTCAAAAATTTTGCGGCTTTGTCGTA
AATTTTTTTCTGGTGGGGCTCATACCCCGCCACTCGAGCGACAGGCGTTTGCCCTCTTCCC
ACAAACCATTCCCTGGGCGCTCATCATCACGTGTCAACCAGTCACTAACCACCTGTCAAT
AGGAAGCCGCCGAGCTCGGTAAGGGTTCCTTCAAGTACGCCTGGGTTCTTGACAAGCTC
AAAGCCGAGCGTGAGCGTGGTATCACCATTGATATCGCCCTCTGGAAGTTCGAGACTCCT
CGCTACTATGTCACCGTCATTGGTATGTTGTCACCACTGCTGTCATCACATTCTCATACTA
ACATGGCTATCAGACGCTCCCGGTCACCGTGATTTCATCAAGAACATGATCACTGGGTAC
TT

Strain No. 15

CGCCACGTCGACTCTGGCAAGTCGACCACTGTGAGTACTACCCACGATGATTTGCTTATC
AGCAGTCATCAACCCCGCCAGATGTGGCGGGGTAATTTCAACTTGAATATTTGCTGACAA
GATTGCATAGACCGGTCACTTGATCTACCAGTGCGGTGGTATCGACAAGCGAACCATCG
AGAAGTTCGAGAAGGTTGGTTTCCATTTCCCCGATCGCACGCCCTCTACCCACCGATCCA
TCAGTCGAATCAGTTACGACGATTGAATATGCGCCTGTTACCCCGCTCGAGTACAAAATT
TTGCGGTTCAACCGTAATTTTTTTTGGTGGGGTTTCAACCCCGCCACTCGAGCGACAGAC
GTTTGCCCTCTTCCCAGAAACCCATGTCTTGTGCATCACGTGTCCATCAGCCACTAACCAC
CCGACAATAGGAAGCCGCCGAGCTCGGTAAGGGTTCCTTCAAGTACGCCTGGGTTCTTG
ACAAGCTCAAGGCTGAGCGTGAGCGTGGTATCACCATCGATATCGCCCTCTGGAAGTTC
GAGACTCCTCGCTACTATGTCACCGTCATTGGTACGTTATCGTCACTTACACTCAATACTT
TCTCATGCTAACATGTACTTCAGACGCTCCCGGTCACCGTGATTTCATCAAGAACATGAT
CACTGGTACTTC

Strain No. 16

ACTCTGGCAAGTCGACCACTGTGAGTACTACCCTCGACGATGGGCTTATCTGCTATCGGA
ACCCTGACCAAGACCTGGCGGGGTATTCCTCTCAAGACAACTTGCTGACATCGCTTGACA
GACCGGTCACTTGATCTACCAGTGCGGTGGTATCGACAAGCGAACCATCGAGAAGTTCG
AGAAGGTTAGTAACTTTCCCTTCGATCGCGCGTCCTTTGTATATCGATTTCCCCTACGACT
CGAAACGTGCCCGCTACCCCGCTCGAGACCAAAAATTTTGCGATATGACCGTAATTTTTT
TGGTGGGGCATTTACCCCGCCACTCGAGCGATGGGCGCGTTTTTGCCCTTTCCTATCCACA
CTTCAATGAGCGCAACATCACGTGTCAAGCAGTCACTAACCATCTGACAATAGGAAGCC
GCTGAGCTCGGTAAGGGTTCCTTCAAGTACGCCTGGGTTCTTGACAAGCTCAAGGCCGAG
CGTGAGCGTGGTATCACCATCGATATTGCTCTCTGGAAGTTCGAGACTCCCCGCTACTAT
GTCACCGTCATTGGTATGTTGTCGCTCACGCCTTACTCTAATTCCTGGTACTAACATGTCT
CTCAGACGCTCCCGGTCACCGTGACTTCATCAAGAACATGATCATGGTTACCTTCCAAAA
A

Strain No. 17

CGTCGACTCTGGCAGTCGACCACTGTGAGTACTACCCTCGACGATGAGCTTATCTGTCAT
CGTGATCCTGACCAAGATCTGGCGGGGTATATCTCAGAAGACAATATGCTGACATCGCTT
CACAGACCGGTCACTTGATCTACCAGTGCGGTGGTATCGACAAGCGAACCATCGAGAAG
TTCGAGAAGGTTAGTCACTTTCCCTTCGATCGCGCGTCCTTTGCCCACCGATTTCCCTTAC
GATTCGAAACGTGCCTGCTACCCCGCTCGAGACCAAAAATTTTGCGATATGACCGTAATT
TTTTTTGGTGGGGCATTTACCCCGCCACTCGAGTGATGGGCGCGTTTTGCCCTTTCCTGTCC
ACAACCTCAATGAGCGCATTGTCACGTGTCAAACTAACCATTCGACAATAGGAAGCCGC
TGAGCTCGGTAAGGGTTCCTTCAAGTACGCCTGGGTTCTTGACAAGCTCAAGGCCGAGCG
TGAGCGTGGTATCACCATCGATATTGCTCTCTGGAAGTTCGAGACTCCTCGCTACTATGTC
ACCGTCATTGGTATGTTGTCGCTCATGCTTCATTCTACTTATTCATACTAACATATCATTCA
GACGCTCCCGGTCACCGTGATTTCATCAAGAACATGATCATGGGTACTTTC

Strain No. 18
CTGGCAAGTCGACCACTGTGAGTACTACCCTTGACGATGAGCTTATCTGCCATCGTAATC
CTGACCAAGATCTGGCGGGGTGTATCTCAAAAGACAACATGCTGACATAGCTTCACAGA
CCGGTCACTTGATCTACCAGTGCGGTGGTATCGACAAGCGAACCATCGAGAAGTTCGAG
AAGGTTAGTCTCCCTTCGAGCGCGCGTCCTTTGCCCATCGATTTTCCCCTACGACTCGAAA
CGTGCCCGCTACCCCGCTCGAGACCAAAAATTTTGCGATATGACCGTAATTTTTTTTGGTG
GGGCATTTACCCCGCCACTCGAGCGATGGGCGCGTTTGCCCTCTCCACAATCTCAATGAG
CGCATCGTCACGTGCCAAGCAGTCACTAACCATCCGACAATAGGAAGCCGCTGAGCTCG
GTAAGGGTTCCTTCAAGTACGCCTGGGTTCTTGACAAGCTCAAGGCCGAGCGTGAGCGTG
GTATCACCATCGATATCGCTCTCTGGAAGTTCGAGACTCCTCGCTACTATGTCACCGTCAT
TGGTATGTTGTCGCCCATGCTTCATTCTTCTTCCTCGTACTAACATATCACTCAGACGCTCC
CGGTCACCGTGATTTCATCAAGAACATGATCACTGGTACTTCCA

Strain No. 19
GGCCACGTCGACTCTGGCAAGTCGACCACTGTGAGTACTACCCTTGACGATGAGCTTATC
GGCCATCGTAAAACCGCCCAAGACCTGGCGGGGTATCTCCCAAAAGAAAACATGCTGAC
ATCGCTTCACAGACCGGTCACTTGATCTACCAGTGTGGTGGTATCGACAAGCGAACCATC
GAGAAGTTCGAGAAGGTTAGTCACAATCCCTTCGATCGCGCGTCCTTTGGCCACCGATTT
CTCCCTACGACTCGAAACGTGCCCGCTACCCCGCTCGAGTCCAAAAATTTTGCGATATGT
CCGTAATTTTTTTGGTGGGGCATTTACCCCGCCACTCCAGCGGCGCGTTTCTGCCCTCTCC
CATTCCACAACCTCACTGAGCGCATTGTCACGTGTCAAGCAGTCACTAACCATTCGACAA
TAGGAAGCCGCTGAGCTCGGTAAGGGTTCCTTCAAGTACGCCTGGGTTCTTGACAAGCTC
AAGGCCGAGCGTGAGCGTGGTATCACCATCGATATCGCTCTCTGGAAGTTCGAGACTCCT
CGCTACTATGTCACCGTCATTGGTATGTTGTGGCTCATGCTCCGTTCTACATCTCTTCTTAC
TAACATATCATTCAGACGCTCCCGGTCACCGTGATTTCATCAAGAACATGATCACTGGGT
TACTT

Strain No. 20
GACTCTGGCAGTCGACCACTGTAAGTACTATCCTCGACGAGCCCGCTTATCTGCACTCGG
CAACCCTGCCTCAGATCTCGCGGGGTATTCTTCAAAGCAACACATGCTGACATACTTTGA
TAGACCGGTCACTTGATCTACCAGTGCGGTGGTATCGACAAGCGAACCATCGAGAAGTT
CGAGAAGGTTAGTCACTTTCCCTTCAATCGCGCGCCCTTCAGCCCATCGATTCCCTTTTCG
AATCCCTCCCAGACGACTCGATCAGCGCCGGCTACCCCGCTCGAGCCCAAAGATTTTGC
GGTTCGACCGTAATTTTTTTTGGTGGGGTATCTACCCCGCCACTAGAGTGACGGGCGCGC
TTGCCCTGTTCCCACAAAACCAAAATGGGCGCGCATCATCACGTGTCAATCAGTCACTAA
CCACTGGACGATAGGAAGCCGCTGAGCTCGGTAAGGGTTCCTTCAAGTACGCCTGGGTC
CTTGACAAGCTCAAGGCCGAGCGTGAGCGTGGTATCACCATCGATATTGCTCTCTGGAAG
TTCGAGACTCCCCGCTACTATGTCACCGTCATTGGTATGTTGCCACTGCTGTCTTGATCTC
GTTCTCATGCTAACATCTCACCCAGACGCTCCCGGTCACCGTGATTTCATCAAGAACATG
ACTGGG

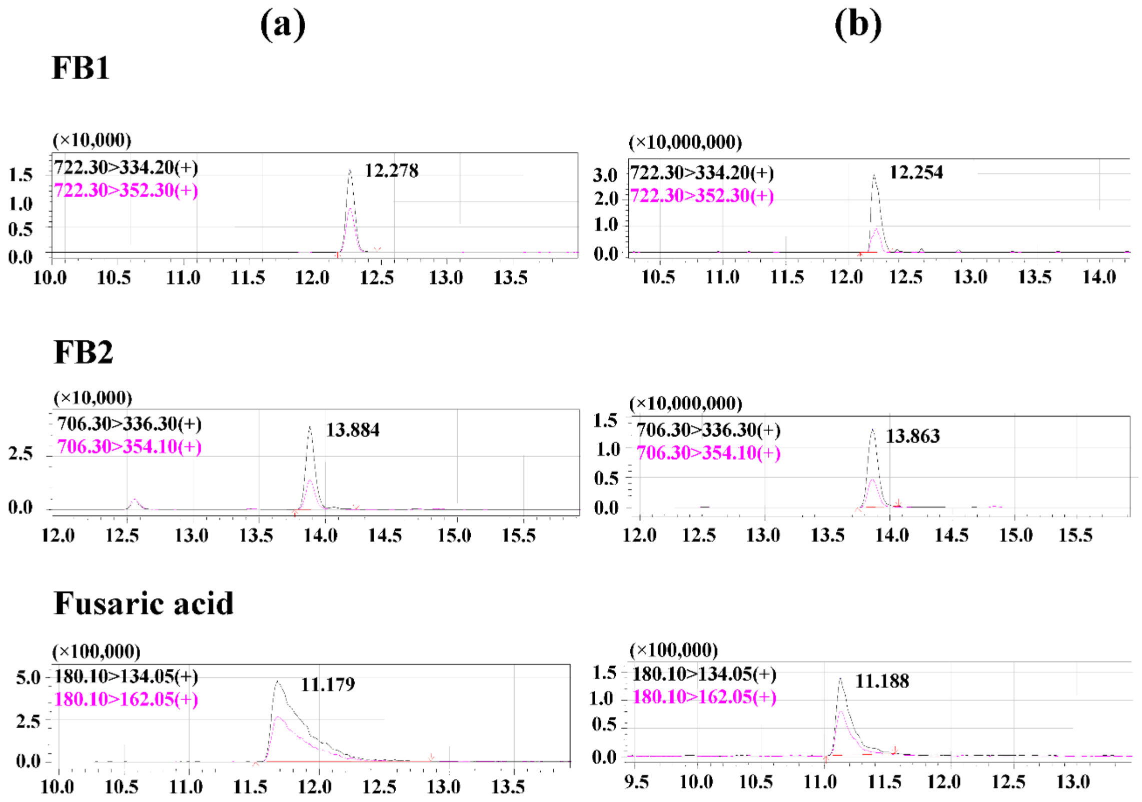

Figure S1. LC-MS/MS chromatogram of FB1, FB2 and fusaric acid ((**a**): standards; (**b**): samples: rice medium, *F. proliferatum*).

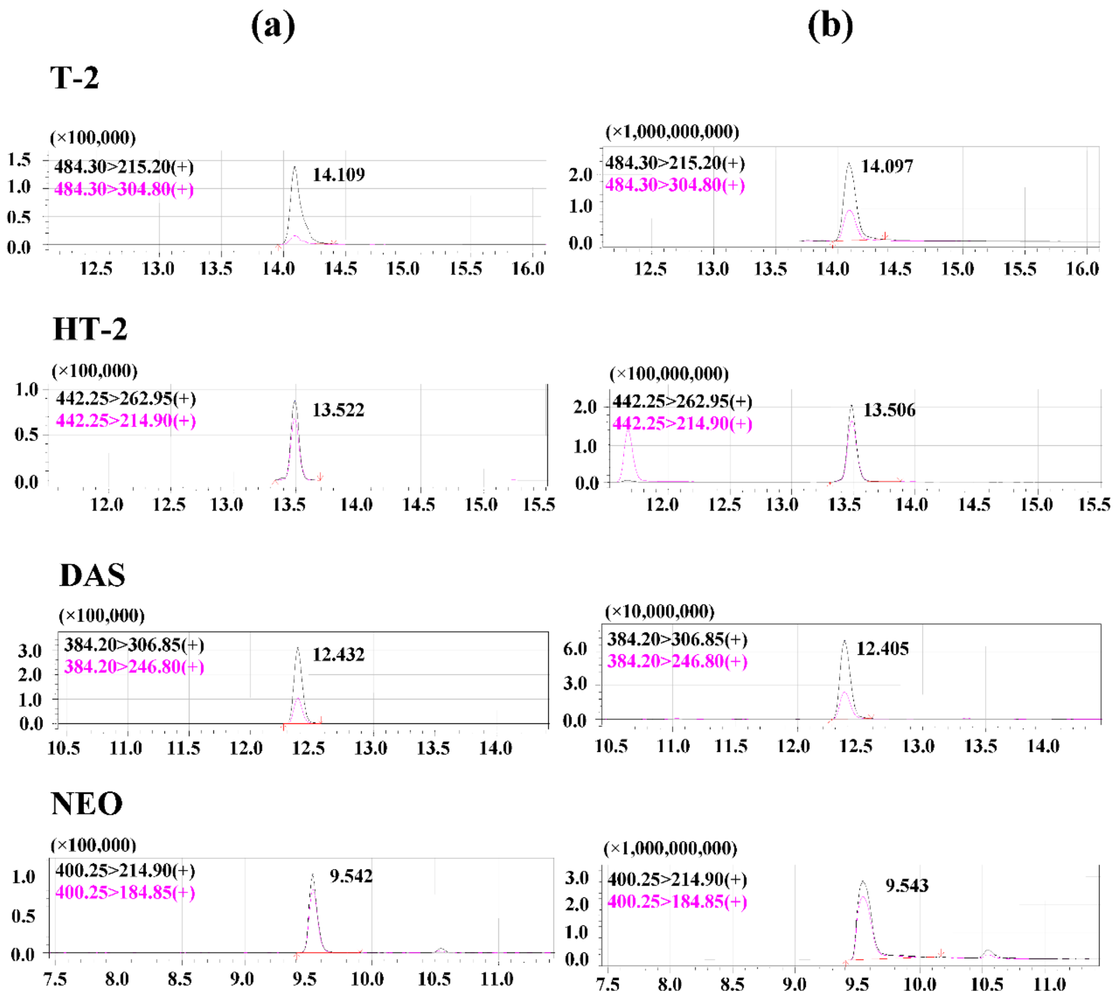

Figure S2. LC-MS/MS chromatogram of four type A trichothecenes ((**a**): standards; (**b**): samples: rice medium, *F. langsethiae*).

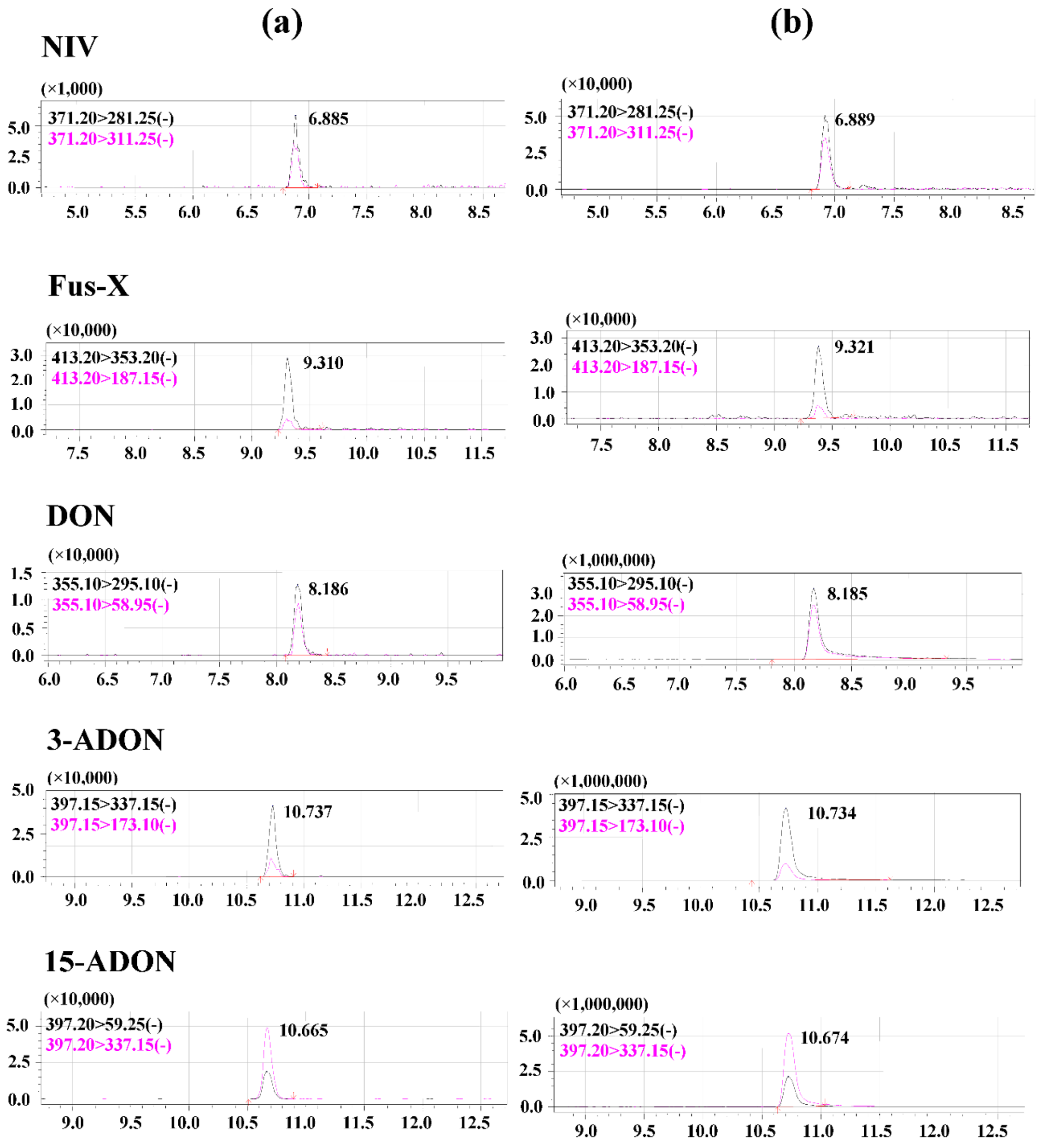

Figure S3. LC-MS/MS chromatogram of five type B trichothecenes ((**a**): standards; (**b**): samples: NIV, Fus-X: maize medium, *F. poae*; DON, ADONs: maize medium, *F. graminearum*).

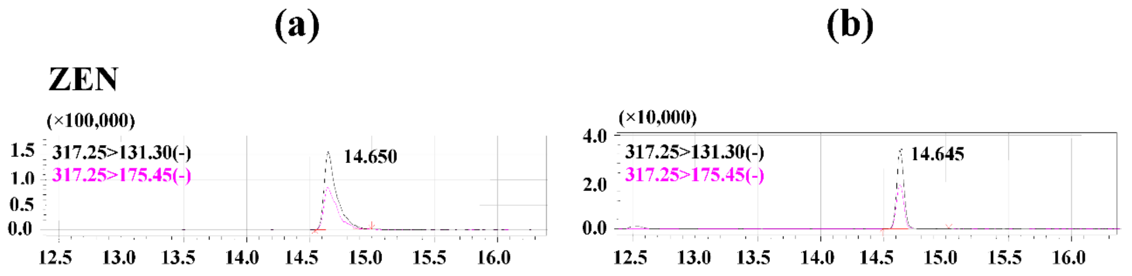

Figure S4. LC-MS/MS chromatogram of ZEN ((**a**): standards; (**b**): samples: rice medium, *F. graminearum*).
